# The Institutional Library

**Published:** 1913-07-05

**Authors:** 


### THE INSTITUTIONAL LIBRARY.

BABIES. By MARGARET FRENCH. (London: Macmillan and Co. 1913. Pp. 80. Price 1s. net.)

WE have referred once or twice already to the extraordinary output of new books on babyhood during the last eighteen months; and several of the new-comers have seemed to us little likely to compete successfully with some of the others. This booklet, however, is decidedly not in that class. For one thing, it is addressed to maternity nurses, not to young mothers; and thus caters for a rather different public from most of the recent baby text-books. For another, it is written out of the fulness of a ripe experience, as anyone can see who troubles to read two or three pages. Briefly, the book is packed full of practical lore such as a maternity nurse might take a lifetime in picking up for herself: common sense is the keynote all through. The sections dealing with the abnormal baby are perhaps a shade less good than those upon the management of the normal infant; but, after all, if every nurse followed Miss French's hints, abnormal babies would be uncommon. If we had to select one chapter rather than another for especial praise, we think perhaps the one which deals with the care of premature babies would get our vote; but there are others which run it very close.

THE PARENTS' BOOK: A BOOK WHICH ANSWERS CHILDREN'S QUESTIONS. By RITA STRAUSS, assisted by a Staff of Contributors. (London: T. C. and E. C. Jack. 1913. Pp. 750. Price 3s. 6d.)

A WONDERFULLY cheap book and a reference library in itself. This is the impression which "The Parents' Book" leaves behind. We can imagine that those who have to deal with children will greet this publication with a sense of relief at the prospect that therein innumerable awkward posers will be solved. Answers are given to thousands of questions on every conceivable topic—indoors and outdoors—ships, railways, and other things that "go," telephones, history, zoology, physiology, and so on are all explained. The information is given in simple language and free from discursive detail, so that it may be easily remembered. There are about a dozen sections in which answers are grouped together—an arrangement much more satisfactory than an interminable alphabetical series. In spite of the very large amount of material at the inquirer's command, no doubt some parents will still be "floored" even though they seek its pages, but this cannot detract from the opinion that this delightful encyclopædia is both a good investment and a source of lasting interest, even if it does not quite cover all the ground of a child's imagination or curiosity.

